# Ca^2+^ Signaling in T-Cell Subsets with a Focus on the Role of Ca_v_1 Channels: Possible Implications in Therapeutics

**DOI:** 10.3389/fimmu.2013.00150

**Published:** 2013-06-20

**Authors:** Lucette Pelletier, M. Savignac

**Affiliations:** ^1^INSERM U1043, CNRS U5282, Center of Physiopathology from Toulouse Purpan, University Paul SabatierToulouse, France; ^2^European Group of Research (GDRE) Ca^2+^ Toolkit Coded Proteins as Drug Targets in Animal and Plant Cells

The role of voltage-dependent calcium (Ca_v_1) channels is prominent in excitable cells while store-operated calcium channels (SOCC) were considered as characteristic of non-excitable cells. Ca_v_1 channels are implicated in excitation transcription. Store-operated calcium channels (SOCC) activity is increased during cardiac stress and would contribute to Ca^2+^ influx and expression of genes responsible for cardiac hypertrophy and heart failure (Luo et al., 2012). Several lines of evidence now show the importance of Ca_v_1 channels in non-excitable cells including lymphocytes (reviewed in Robert et al., 2011, 2013). Ca_v_1 channels are defined by their voltage sensitivity and their sensitivity to drugs as dihydropyridines, phenylalkylamines, benzothiazepines, known to alter T-cell functions. However the drug concentrations needed were higher compared to excitable cells. The absence of cell membrane depolarization upon activation and possible non-specific effects of the drugs questioned the putative role of Ca_v_1 channels in T-cells.

Ca_v_1 channels are formed by the ion forming pore α1 subunit encoded by four genes conferring some tissue-specific expression pattern in excitable cells. Ca_v_1.1 is characteristic of skeletal muscle cells. Ca_v_1.2 is found in neurons, heart, and smooth muscle cells while Ca_v_1.3 is detected in neuroendocrine cells. Ca_v_1.2 and Ca_v_1.3 can be found in the same tissues even if their role is not redundant as shown by the differential phenotypes of Ca_v_1.2 and Ca_v_1.3 null mice. Ca_v_1.4 is the retinal form. Ca_v_1 channel isoforms differ by their sensitivity to depolarization and to antagonizing drugs such as dihydropyridines (DHP) as well as by their inactivation properties (Lipscombe et al., 2004). For example, Ca_v_1.4 channels activate at more negative potentials than Ca_v_1.3 and Ca_v_1.2, which highlights the potential involvement of Ca_v_1.4 in non-excitable cells as mast cells (McRory et al., 2004) and more recently in mouse T-lymphocytes (Omilusik et al., 2011).

## Calcium in T-Lymphocytes: Prominent Role of the Stim-Orai Pathway

In T-lymphocytes, Ca^2+^ ions are important for the activation of many enzymes including phospholipase C gamma (PLCγ), classical protein kinases C, for proper protein folding, for the accessibility of key enzymes in T-cell transduction, and as a second messenger (Vig and Kinet, 2009). Variations in the intracellular calcium concentration ([Ca]_i_) are responsible for modulating the transcription of more than 75% of genes induced or down-regulated by T-cell receptor engagement in T-lymphocytes (Feske et al., 2001). The intracellular [Ca]_i_ that decides the cellular fate is tightly regulated in both resting and activated conditions. The calcium concentration in the external medium is about 1–2 mM, whereas the [Ca]_i_ is about 50–100 nM and depends on the calcium channels expressed at both the cell and endoplasmic reticulum (ER) membranes, on exchangers, pumps, … Activation of potassium channels that extrude the potassium from the cell is needed for supporting the electrochemical driving force allowing the calcium influx. In T-lymphocytes, TCR engagement results in a cascade of tyrosine kinase activation, the constitution of a platform transducing the signal with the recruitment of adapters and enzymes such as PLCγ that generates inositol trisphosphate (IP3) and diacylglycerol. IP3 binds to its receptors on the ER membrane leading to the release of ER Ca^2+^ stores, which induces a conformational change of STIM1, an ER Ca^2+^ sensor. STIM1 then localizes near the cell membrane, and activates the SOCC ORAI1 at the cell membrane (Barr et al., 2009; Oh-hora, 2009; Vig and Kinet, 2009; Zhou et al., 2010). The sustained entry of Ca^2+^ into the cell through ORAI channels is responsible for the activation of calcineurin, resulting in the nuclear translocation of the transcription factor NFAT as well as the activation of calmodulin kinase-dependent pathways. The severe immunodeficiency observed in mice or Humans with defective STIM1 (Picard et al., 2009) and ORAI1 testifies the importance of these molecules in T-cell biology (Partiseti et al., 1994; Feske et al., 2006, 2012).

However, this scheme accounts neither for the heterogeneity of calcium responses induced by TCR stimulation depending upon the state of activation and differentiation of T-lymphocytes nor for the possible implication of other calcium channels at the T-cell membrane.

## Ca_v_1 Channels in T-Cells

**Figure 1:**
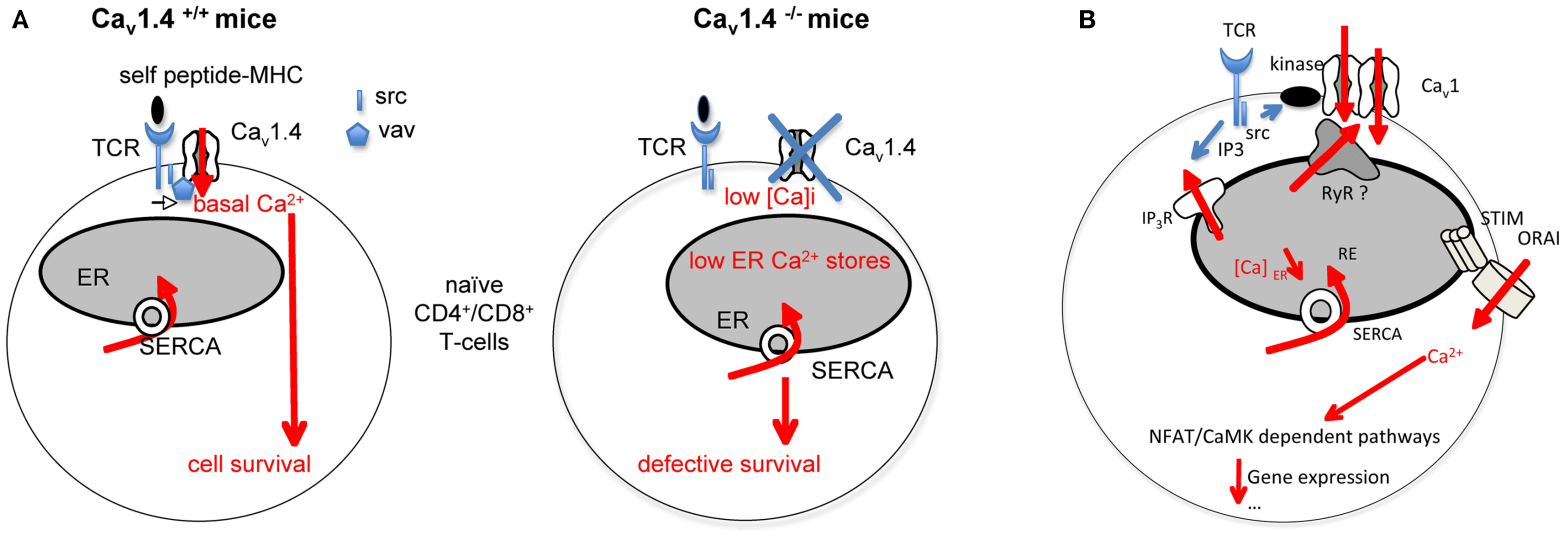
**Role of Ca_v_1 channels in T-cell Ca^2+^ responses and functions**. **(A)** Ca_v_1.4 is found localized in preformed complexes containing src kinase and Vav. Self peptide-MHC interactions with the TCR, independently of the antigen specificity would induce a survival signal in naïve T-cells. This signal requires some calcium entry depending upon the Ca_v_1.4 containing complex. Ca_v_1.4 would be also important for maintaining correct endoplasmic reticulum (ER) Ca^2+^ stores. Ca_v_1.4 null T-cells exhibit defective calcium homeostasis associated with defective survival. **(B)** The scheme depicts how we assume Ca_v_1.2 channel regulation in Th2-cells. TCR activation would lead to src and PKC enzyme activation. Possible PKC-Ca_v_1.2 interactions would induce Ca_v_1.2 channel opening. Ca_v_1.2 channels can interact with Ryanodine receptors (RyR) at the membrane of the endoplasmic reticulum (ER). These channels release Ca^2+^ from the ER into the cytosol. The depletion of ER Ca^2+^ stores would allow conformational changes of the Ca^2+^ sensor STIM and the subsequent activation of ORAI channels. IP3R, IP3 receptors; MHC, major histocompatibility complex; SERCA, sarco/endoplasmic reticulum Ca^2+^ ATPase; TCR, T-cell receptor.

An increasing line of evidence pleads for the involvement of Ca_v_1 channels in T-lymphocyte biology (Kotturi et al., 2003, 2006; Stokes et al., 2004; Kotturi and Jefferies, 2005; Badou et al., 2006; Matza et al., 2008, 2009; Jha et al., 2009). Thus, the analysis of mice with ablation of the auxiliary subunits Ca_v_β3 (Jha et al., 2009) and Ca_v_β4 (Badou et al., 2006) and more recently of mice deleted for Ca_v_1.4 (Omilusik et al., 2011) reveals the role of Ca_v_1 channels in T-lymphocyte survival and activation. Ca_v_1.4 was recently described as interacting with Vav and lck src kinase (Jha et al., 2009), which could result in Ca^2+^ entry required for maintaining [Ca]_i_ and the ER Ca^2+^ stores (Figure 1A). As a consequence, Ca_v_1.4 defective T-cells are more prone to apoptosis and have a reduced homeostatic proliferation capacity. Naïve Ca_v_1.4 null T-cells also harbor defective calcium influx upon TCR stimulation suggesting the involvement of these channels in TCR-dependent Ca^2+^ signaling (Omilusik et al., 2011). Interestingly, the human Timothy syndrome which is associated to mutation in gene encoding for Ca_v_1.2 resulting in excessive Ca^2+^ entry is associated in most patients with an immunosuppression suggesting a role for Ca_v_1.2 channels in immune functions (Liao and Soong, 2010). It will be interesting to determine if and how the Ca_v_1.2 mutation affects immune cell functions.

## Ca_v_1 Channels in Th2-Cells

Depending upon the strength of TCR stimulation, the chronicity of antigenic exposure, the route of antigen administration, and the cytokines present during T-cell differentiation, CD4^+^ T-cells can differentiate into Th1, Th2, and Th17-cells that produce distinct sets of cytokines and exert different functions. In addition, these subpopulations express lineage specific and common transcription factors. Th1-cells produce gamma interferon (IFN-γ) and are implicated in the eradication of intracellular pathogens, viruses; Th2-cells produce interleukin (IL)-4, IL-5, and IL-13, contribute to the elimination of parasites and Th17, producing IL-17 and IL-22, participate in the elimination of extracellular pathogens as fungi. These subsets may also be pathogenic. Th1 and Th17 can promote autoimmune diseases, whereas Th2-cells can cause allergic diseases. Especially, Th2-cells can induce all the cardinal features of allergic asthma through all the cytokines they produce.

The calcium signature differs between Th1, Th2, and Th17-cells suggesting that components regulating calcium entry may differ between each T-cell subsets. The resting [Ca]i is the lowest in Th1, the highest in Th2, and intermediate in Th17. Conversely the TCR-dependent increase in [Ca]i is the highest in Th1, intermediate in Th17, and less important and sustained in Th2-cells, which could be related to the differential dependence of calcium-regulated transcription factors as NFAT, NFkB, and CREB (Dolmetsch et al., 1997) in the different T-cell subsets. It was suggested that these differences could result from lower equipment in pumps or in potassium channels required for maintaining the electrochemical driving force that supports calcium entry in Th2-cells, compared with the other T-cell subsets (Fanger et al., 2000). Our group identified voltage-dependent calcium Ca_v_1.2 and Ca_v_1.3 channels as selectively overexpressed in Th2-cells (Badou et al., 1997; Savignac et al., 2001, 2004; Gomes et al., 2006; Djata Cabral et al., 2010). Knocking down Ca_v_1.2 and/or Ca_v_1.3 α1 subunits by transfection with specific antisense oligodeoxynucleotides (Ca_v_1AS) did not affect the proliferative response of Th2-cells but strongly impaired the TCR-dependent increase in [Ca]_i_ and Th2 cytokine production without any effect on Th1-cells. We have then injected OVA-specific DO11.10 transgenic Th2-cells transfected or not with Ca_v_1.2 plus Ca_v_1.3 AS into BALB/c mice that were given intranasal OVA. Th2 Ca_v_1AS localized into the lungs and proliferated as well as control Th2-cells. However they were unable to support a sustained inflammation characteristic of asthma. On the contrary, Th1 Ca_v_1AS were as effective as control Th1-cells in the induction of inflammation. Antisense oligodeoxynucleotides were shown to remain localized into the airways when given by inhalation (Tanaka and Nyce, 2001). A mixture of Ca_v_1.2 and Ca_v_1.3 AS given by this route protected mice against the development of asthma (Djata Cabral et al., 2010), suggesting that these channels may represent an interesting new approach in the treatment of allergic diseases. Interestingly TCR stimulation is associated with polarized signaling as shown by an enrichment of Ca^2+^ (Lioudyno et al., 2008) and other ionic channels near the immune synapse, an area where the T-cell membrane contacts the antigen-presenting cell (Cahalan and Chandy, 2009). It will be important to assess whether Ca_v_1 channels traffic at the immune synapse upon TCR activation, in which areas and to identify the partners with which they associate.

Another important feature of the regulation of [Ca]_i_ will be the understanding of ionic channels as potassium and non-specific cationic channels in Ca_v_1 channel opening. Indeed K^+^ channels comprising voltage and Ca^2+^-activated channels maintain the electrochemical gradient of Ca^2+^ required for Ca^2+^ entry and tend to hyperpolarize the cell membrane favoring Ca_v_1 channel inactivation. Conversely, TCR-dependent TRPM4 activation induces Na^+^ entry described as limiting the Ca^2+^ entry and permitting Ca^2+^ oscillations (Launay et al., 2004). It would be interesting to determine if TRPM4 can favor Ca_v_1 channel opening.

## Regulation of Ca_v_1 Channels in Lymphocytes

All the authors showing the presence of Ca_v_1 channels in T-lymphocytes agree that these channels are not voltage-operated in physiological conditions. Therefore, how they are regulated in T-lymphocytes must be explained. Differences in the sequence/structure of Ca_v_1 channels in T-cells relative to the canonical forms in excitable cells have been reported and could provide an account for the absence of voltage sensitivity in T-cells (Stokes et al., 2004; Kotturi and Jefferies, 2005). However, the authors must demonstrate that the truncated Ca_v_1 channels are true Ca^2+^ channels. The analysis of T-lymphocytes from Ca_v_1.4 null and sufficient mice reveals the presence of voltage-gated currents in control cells, which were undetectable in null T-cells (Omilusik et al., 2011). Noticeably, the authors used peculiar conditions for their patch clamp experiments and mentioned that normal T-cells were pre-activated before recordings. This suggests that TCR activation could induce or enhance the number of Ca_v_1 channels at the cell membrane. The TCR-induced opening of Ca_v_1 channels may alternatively be explained by the existence of partners able to drive channel recruitment via two non-exclusive pathways: (i) post-translational modifications regulating Ca_v_1.2 channel availability at the cell membrane without voltage change and/or (ii) modification of channel trafficking, targeting, recycling, or degradation induced by TCR stimulation. We demonstrate that the sequence of Ca_v_1 channels in Th2-lymphocytes is similar to neuronal forms of the channel. However, Ca_v_1 channels do not seem to be voltage-operated in Th2-lymphocytes. We have already demonstrated that TCR-induced L-type dependent calcium influx is at least sensitive to Src kinases and the PKC in an IL-4 producing T-cell hybridoma (Savignac et al., 2001). In fact, the application of PP2, an inhibitor of Src kinases or an inhibitor of PKCα on Th2-cells suppresses the Ca_v_1 channel-dependent Ca^2+^ influx. In addition, we showed that PKC activator induced an entry of Ca^2+^, suppressed by an antagonist of Ca_v_1 channels (Savignac et al., 2001). These data mean that kinase activation is implicated in Ca_v_1 dependent currents (Figure 1B). PKCα is a good candidate since Ca_v_1.2 channels can be constitutively activated at the resting potential of smooth arteriolar cells due to their interaction with PKCα (Navedo et al., 2005; Santana and Navedo, 2010). Ryanodine receptors (RyR) are channels releasing Ca^2+^ from the ER into the cytosol. They are activated directly or not by Ca_v_1 channels. It is not known if Ca_v_1 channels interact with RyR in T-lymphocytes, inducing ER Ca^2+^ depletion and the activation of the STIM-ORAI pathway (Figure 1B).

The pending questions deal with how Ca_v_1 channels work in lymphocytes and their integration with other channels to generate a specific calcium signature. The relationships between STIM, ORAI, and Ca_v_1 are puzzling. STIM was shown as a negative regulator of Ca_v_1 signaling (Park et al., 2010). The possibility of a checkpoint controlling ORAI versus Ca_v_1 channel-dependent calcium responses merits to be explored.

## References

[B1] Badou A. Jha M. K. Matza D. Mehal W. Z. Freichel M. Flockerzi V. (2006). Critical role for the beta regulatory subunits of Cav channels in T lymphocyte function. Proc. Natl. Acad. Sci. U.S.A. 103, 15529–15534 10.1073/pnas.0607262103 17028169 PMC1622857

[B2] Badou A. Savignac M. Moreau M. Leclerc C. Pasquier R. Druet P. (1997). HgCl_2_-induced IL-4 gene expression in T cells involves protein kinase C-dependent calcium influx through L-type calcium channels. J. Biol. Chem. 272, 32411–32418 10.1074/jbc.272.51.32411 9405450

[B3] Barr V. A. Bernot K. M. Shaffer M. H. Burkhardt J. K. Samelson L. E. (2009). Formation of STIM and Orai complexes: puncta and distal caps. Immunol. Rev. 231, 148–159 10.1111/j.1600-065X.2009.00812.x 19754895 PMC3110759

[B4] Cahalan M. D. Chandy K. G. (2009). The functional network of ion channels in T lymphocytes. Immunol. Rev. 231, 59–87 10.1111/j.1600-065X.2009.00816.x 19754890 PMC3133616

[B5] Djata Cabral M. Paulet P. E. Robert B. Gomes M. L. Renoud M. Savignac C. (2010). Knocking-down Cav1 calcium channels implicated in Th2-cell activation prevents experimental asthma. Am. J. Respir. Crit. Care Med. 181, 1310–1317 10.1164/rccm.200907-1166OC 20167851

[B6] Dolmetsch R. E. Lewis R. S. Goodnow C. C. Healy J. I. (1997). Differential activation of transcription factors induced by Ca2+ response amplitude and duration. Nature 386, 855–858 10.1038/386855a0 9126747

[B7] Fanger C. M. Neben A. L. Cahalan M. D. (2000). Differential Ca2+ influx, KCa channel activity, and Ca^2+^ clearance distinguish Th1 and Th2 lymphocytes. J. Immunol. 164, 1153–1160 10640725 10.4049/jimmunol.164.3.1153

[B8] Feske S. Giltnane J. Dolmetsch R. Staudt L. M. Rao A. (2001). Gene regulation mediated by calcium signals in T lymphocytes. Nat. Immunol. 2, 316–324 10.1038/86318 11276202

[B9] Feske S. Gwack Y. Prakriya M. Srikanth S. Puppel S. H. Tanasa B. (2006). A mutation in Orai1 causes immune deficiency by abrogating CRAC channel function. Nature 441, 179–185 10.1038/nature04702 16582901

[B10] Feske S. Skolnik E. Y. Prakriya M. (2012). Ion channels and transporters in lymphocyte function and immunity. Nat. Rev. Immunol. 12, 532–547 10.1038/nri3233 22699833 PMC3670817

[B11] Gomes B. Savignac M. Cabral M. D. Paulet P. Moreau M. Leclerc C. (2006). The cGMP/protein kinase G pathway contributes to dihydropyridine-sensitive calcium response and cytokine production in TH2 lymphocytes. J. Biol. Chem. 281, 12421–12427 10.1074/jbc.M510653200 16533816

[B12] Jha M. K. Badou A. Meissner M. McRory J. E. Freichel M. Flockerzi V. (2009). Defective survival of naive CD8^+^ T lymphocytes in the absence of the beta3 regulatory subunit of voltage-gated calcium channels. Nat. Immunol. 10, 1275–1282 10.1038/ni.1793 19838200 PMC2785134

[B13] Kotturi M. F. Carlow D. A. Lee J. C. Ziltener H. J. Jefferies W. A. (2003). Identification and functional characterization of voltage-dependent calcium channels in T lymphocytes. J. Biol. Chem. 278, 46949–46960 10.1074/jbc.M309268200 12954628

[B14] Kotturi M. F. Hunt S. V. Jefferies W. A. (2006). Roles of CRAC and Ca(V)-like channels in T cells: more than one gatekeeper? Trends Pharmacol. Sci. 27, 360–367 10.1016/j.tips.2006.05.007 16766050

[B15] Kotturi M. F. Jefferies W. A. (2005). Molecular characterization of L-type calcium channel splice variants expressed in human T lymphocytes. Mol. Immunol. 42, 1461–1474 10.1016/j.molimm.2005.01.014 15899519

[B16] Launay P. Cheng H. Srivatsan S. Penner R. Fleig A. Kinet J. P. (2004). TRPM4 regulates calcium oscillations after T cell activation. Science 306, 1374–1377 10.1126/science.1098845 15550671

[B17] Liao P. Soong T. W. (2010). CaV1.2 channelopathies: from arrhythmias to autism, bipolar disorder, and immunodeficiency. Pflugers Arch. 460, 353–359 10.1007/s00424-009-0753-0 19916019

[B18] Lioudyno M. I. Kozak J. A. Penna A. Safrina O. Zhang S. L. Sen D. (2008). Orai1 and STIM1 move to the immunological synapse and are up-regulated during T cell activation. Proc. Natl. Acad. Sci. U.S.A. 105, 2011–2016 10.1073/pnas.0706122105 18250319 PMC2538873

[B19] Lipscombe D. Helton T. D. Xu W. (2004). L-type calcium channels: the low down. J. Neurophysiol. 92, 2633–2641 10.1152/jn.00486.2004 15486420

[B20] Luo X. Berdymammet H. Jiang N. Wang Z. V. Tandan S. Rakalin A. (2012). STIM1-dependent store-operated Ca2+ entry is required for pathological cardiac hypertrophy. J. Mol. Cell. Cardiol. 52, 136–147 10.1016/j.yjmcc.2011.11.003 22108056 PMC3247164

[B21] Matza D. Badou A. Jha M. K. Willinger T. Antov A. Sanjabi S. (2009). Requirement for AHNAK1-mediated calcium signaling during T lymphocyte cytolysis. Proc. Natl. Acad. Sci. U.S.A. 106, 9785–9790 10.1073/pnas.0902844106 19497879 PMC2701053

[B22] Matza D. Badou A. Kobayashi K. S. Goldsmith-Pestana K. Masuda Y. Komuro A. (2008). A scaffold protein, AHNAK1, is required for calcium signaling during T cell activation. Immunity 28, 64–74 10.1016/j.immuni.2007.11.020 18191595 PMC2350190

[B23] McRory J. E. Hamid J. Doering C. J. Garcia E. Parker R. Hamming K. (2004). The CACNA1F gene encodes an L-type calcium channel with unique biophysical properties and tissue distribution. J. Neurosci. 24, 1707–1718 10.1523/JNEUROSCI.4846-03.2004 14973233 PMC6730460

[B24] Navedo M. F. Amberg G. C. Votaw V. S. Santana L. F. (2005). Constitutively active L-type Ca2+ channels. Proc. Natl. Acad. Sci. U.S.A. 102, 11112–11117 10.1073/pnas.0500360102 16040810 PMC1180225

[B25] Oh-hora M. (2009). Calcium signaling in the development and function of T-lineage cells. Immunol. Rev. 231, 210–224 10.1111/j.1600-065X.2009.00819.x 19754899

[B26] Omilusik K. Priatel J. J. Chen X. Wang Y. T. Xu H. Choi K. B. (2011). The Ca(v)1.4 calcium channel is a critical regulator of T cell receptor signaling and naive T cell homeostasis. Immunity 35, 349–360 10.1016/j.immuni.2011.07.011 21835646

[B27] Park C. Y. Shcheglovitov A. Dolmetsch R. (2010). The CRAC channel activator STIM1 binds and inhibits L-type voltage-gated calcium channels. Science 330, 101–105 10.1126/science.1191027 20929812

[B28] Partiseti M. Le Deist F. Hivroz C. Fischer A. Korn H. Choquet D. (1994). The calcium current activated by T cell receptor and store depletion in human lymphocytes is absent in a primary immunodeficiency. J. Biol. Chem. 269, 32327–32335 7798233

[B29] Picard C. McCarl C. A. Papolos A. Khalil S. Luthy K. Hivroz C. (2009). STIM1 mutation associated with a syndrome of immunodeficiency and autoimmunity. N. Engl. J. Med. 360, 1971–1980 10.1056/NEJMoa0900082 19420366 PMC2851618

[B30] Robert V. Triffaux E. Savignac M. Pelletier L. (2011). Calcium signalling in T-lymphocytes. Biochimie 93, 2087–2094 10.1016/j.biochi.2011.06.016 21712067

[B31] Robert V. Triffaux E. Savignac M. Pelletier L. (2013). Singularities of calcium signaling in effector T-lymphocytes. Biochim. Biophys. Acta. 1833, 1595–1602 10.1016/j.bbamcr.2012.12.001 23266355

[B32] Santana L. F. Navedo M. F. (2010). Natural inequalities: why some L-type Ca2+ channels work harder than others. J. Gen. Physiol. 136, 143–147 10.1085/jgp.200910391 20660657 PMC2912067

[B33] Savignac M. Badou A. Moreau M. Leclerc C. Guery J. C. Paulet P. (2001). Protein kinase C-mediated calcium entry dependent upon dihydropyridine sensitive channels: a T cell receptor-coupled signaling pathway involved in IL-4 synthesis. FASEB J. 15, 1577–1579 11427491 10.1096/fj.00-0733fje

[B34] Savignac M. Gomes B. Gallard A. Narbonnet S. Moreau M. Leclerc C. (2004). Dihydropyridine receptors are selective markers of Th2 cells and can be targeted to prevent Th2-dependent immunopathological disorders. J. Immunol. 172, 5206–5212 15100258 10.4049/jimmunol.172.9.5206

[B35] Stokes L. Gordon J. Grafton G. (2004). Non-voltage-gated L-type Ca^2+^ channels in human T cells: pharmacology and molecular characterization of the major alpha pore-forming and auxiliary beta-subunits. J. Biol. Chem. 279, 19566–19573 10.1074/jbc.M401481200 14981074

[B36] Tanaka M. Nyce J. W. (2001). Respirable antisense oligonucleotides: a new drug class for respiratory disease. Respir. Res. 2, 5–9 10.1186/rr153 11686859 PMC59563

[B37] Vig M. Kinet J. P. (2009). Calcium signaling in immune cells. Nat. Immunol. 10, 21–27 10.1038/ni.f.220 19088738 PMC2877033

[B38] Zhou Y. Meraner P. Kwon H. T. Machnes D. Masatsugu O. H. Zimmer J. (2010). STIM1 gates the store-operated calcium channel ORAI1 in vitro. Nat. Struct. Mol. Biol. 17, 112–116 10.1038/nsmb.1724 20037597 PMC2902271

